# Eye Tracking Research on the Influence of Spatial Frequency and Inversion Effect on Facial Expression Processing in Children with Autism Spectrum Disorder

**DOI:** 10.3390/brainsci12020283

**Published:** 2022-02-18

**Authors:** Kun Zhang, Yishuang Yuan, Jingying Chen, Guangshuai Wang, Qian Chen, Meijuan Luo

**Affiliations:** 1 National Engineering Research Center for E-Learning, Faculty of Artificial Intelligence in Education, Central China Normal University, Wuhan 430079, China; zhk@mail.ccnu.edu.cn (K.Z.); yys1123@foxmail.com (Y.Y.); paviqchen@mails.ccnu.edu.cn (Q.C.); luomeijuan@mails.ccnu.edu.cn (M.L.); 2 National Engineering Laboratory for Educational Big Data, Faculty of Artificial Intelligence in Education, Central China Normal University, Wuhan 430079, China; 3 School of Computer Science, Wuhan University, Wuhan 430072, China; wangguang_shuai@163.com

**Keywords:** autism spectrum disorder, facial expression processing, eye tracking, spatial frequency, inversion effect

## Abstract

Facial expression processing mainly depends on whether the facial features related to expressions can be fully acquired, and whether the appropriate processing strategies can be adopted according to different conditions. Children with autism spectrum disorder (ASD) have difficulty accurately recognizing facial expressions and responding appropriately, which is regarded as an important cause of their social disorders. This study used eye tracking technology to explore the internal processing mechanism of facial expressions in children with ASD under the influence of spatial frequency and inversion effects for improving their social disorders. The facial expression recognition rate and eye tracking characteristics of children with ASD and typical developing (TD) children on the facial area of interest were recorded and analyzed. The multi-factor mixed experiment results showed that the facial expression recognition rate of children with ASD under various conditions was significantly lower than that of TD children. TD children had more visual attention to the eyes area. However, children with ASD preferred the features of the mouth area, and lacked visual attention and processing of the eyes area. When the face was inverted, TD children had the inversion effect under all three spatial frequency conditions, which was manifested as a significant decrease in expression recognition rate. However, children with ASD only had the inversion effect under the LSF condition, indicating that they mainly used a featural processing method and had the capacity of configural processing under the LSF condition. The eye tracking results showed that when the face was inverted or facial feature information was weakened, both children with ASD and TD children would adjust their facial expression processing strategies accordingly, to increase the visual attention and information processing of their preferred areas. The fixation counts and fixation duration of TD children on the eyes area increased significantly, while the fixation duration of children with ASD on the mouth area increased significantly. The results of this study provided theoretical and practical support for facial expression intervention in children with ASD.

## 1. Introduction

Autism spectrum disorder (ASD) is a developmental disability that can cause significant social, communication, and behavioral challenges [1]. The symptoms of ASD begin in early childhood and typically last a lifetime, placing a heavy burden on families and society. The incidence of ASD has been increasing in recent years, with an estimated 1 in 44 children diagnosed with the disorder, according to the Centers for Disease Control of the USA [2]. Facing the growing demand for diagnosis and treatment, more and more research has been carried out [3], and in-depth exploration of the characteristics of social disorders in children with ASD has become one of the research hotspots [4].

Facial expressions play an important role in social interaction and communication. They provide a way to exchange rich social information, and a window for understanding the internal emotional states of others [5]. Good facial expression processing ability, that is, the ability to accurately recognize facial expressions and respond appropriately, is the key to successful communication and social interaction, and is of great significance to individual’s social cognitive development [6]. Typical developing (TD) children are sensitive to facial expression information. They can distinguish partial expressions such as happiness as early as 7 months old [7]. Their ability to accurately recognize facial expressions stabilizes with age. However, many researchers have shown that children with ASD have certain disorders in the processing of facial expressions. They have difficulty in accurately recognizing facial expressions and responding appropriately [8]. This is regarded as one of the important or even core causes of their social disorders [9]. Therefore, in-depth exploration of the internal mechanism of facial expression processing in children with ASD has important research value for improving their social disorders.

Facial expression processing refers to the cognitive process of obtaining facial expression information through vision and then interpreting and understanding it [10]. The ability to process facial expressions mainly depends on whether the facial features related to expressions can be fully acquired, and whether the appropriate processing methods and strategies can be adopted according to different conditions, which is finally reflected in such indicators as the facial expression recognition rate.

There is evidence that different facial areas contain different feature information relevant for different expressions [11]. Many researchers in psychology have demonstrated the importance of eyes and mouth areas in facial expression processing [12]. The Facial Action Coding System proposed by Ekman et al. [13,14] breaks down the facial expressions into individual components of muscle movement, called action units (AUs). One AU or a combination of multiple AUs describe a specific facial expression, including basic expressions and complex expressions [15]. Most of the AUs related to expressions are concentrated around the eyes and mouth. Therefore, the areas of eyes and mouth are rich in facial expression features and are considered to be the core areas for facial expression processing. Exploring whether individuals can pay enough attention to these core areas and acquire sufficient expression information is helpful to analyze their facial expression processing ability.

Many researchers have used eye tracking technology to analyze the visual attention characteristics of children with ASD to the face and specific areas of interest [16], revealing their tacit emotional and cognitive processing. Dalton et al. [17] found that the fixation duration of children with ASD on the mouth was significantly more than that on the eyes. He et al. [18] found that compared with TD children, children with ASD exhibited atypical gaze patterns in facial expression processing tasks. They reduced visual attention to the face, especially the eyes. However, there are also inconsistent conclusions. Wagner et al. [19] found that individuals with ASD relied on the eyes to recognize facial expressions like TD individuals. Lahaie et al. [20] found that children with ASD did not show a gaze preference for the mouth. In addition, some research has shown that individuals with ASD could adjust their processing strategies according to the changes in different facial features and would consciously pay attention to the mouth when facial information was weakened [21]. Therefore, it is necessary to further explore the eye tracking characteristics of children with ASD on the core areas (eyes and mouth) in the facial expression processing, as well as the changes in their visual strategies under different conditions.

The processing methods of visual information mainly include two types: configural/holistic processing and featural/local processing. Configural processing is a processing strategy that processes the spatial configuration of each component and perceives it as a meaningful whole. Featural processing is a processing strategy that only processes local features from a fragmented perspective [22]. Many researchers have shown that there are differences in processing methods between children with ASD and TD children. For example, children with ASD performed better than TD children on embedded figures tests [23], suggesting that they prefer local rather than holistic processing method. The Weak Central Coherence theory proposed by Frith and Happé provides a certain explanation [24]. TD children usually process information at the expense of ignoring local details to form meaning and gestalt configuration. The face is just a gestalt representation integrated by various local features such as eyes, mouth, etc., which is considered a typical case of configural processing [25]. TD children tend to perceive and process the face as a whole. However, children with ASD tend to interpret multiple complex stimuli as independent parts, and then independently perceive and process these local features. It is difficult for them to integrate the local features into a meaningful whole, and perform holistic facial processing like TD children [26].

Researchers have designed many methods, such as spatial frequency paradigm and inverted paradigm, to further experimentally analyze the internal processing mechanism of children with ASD. These paradigms include:

1. The spatial frequency paradigm mainly uses different spatial filters to transform facial expression images [27]. The change of spatial frequency would cause the change of expression features in the facial image, which would have an impact on different facial expression processing methods. It is generally believed that after the low spatial frequency (LSF) filter blurs the facial image, the configural information of the face is retained, which is beneficial to the configural processing method. The high spatial frequency (HSF) filter highlights the local features of the face, which is beneficial to the featural processing method. Additionally, the broad spatial frequency (BSF) is the original image itself [28]. Exploring the performance of individuals under different spatial frequency conditions is helpful to analyze their facial expression processing methods.

Some researchers presented facial expression images under different spatial frequency conditions, and asked the participants to make recognition judgments [29]. Their research found that TD individuals processed facial information more effectively under the LSF condition than the HSF condition, indicating that they mainly adopted the configural processing method. Deruelle et al. [30] first used this paradigm to find that children with ASD were weaker than TD children in recognizing various expressions, and generally relied on HSF information to process facial expressions, confirming that they mainly used featural processing method. However, some researchers believed that changes in spatial frequency would not directly affect the facial expression recognition of individuals with ASD. Vanmarcke et al. [28] found that teenagers with ASD performed worse than TD teenagers with the same age in facial expression classification tasks, but the level of spatial frequency did not significantly affect the performance of these two groups. Goffaux et al. [31] found that although featural information was enhanced under the HSF condition, it still retained certain configural information and still supported the occurrence of configural processing. Therefore, simply presenting facial images with different spatial frequencies does not provide a good insight into how children with ASD process facial expressions.

2. The inversion paradigm adopts the method of inverting the entire facial image, and then asks the participants to perceive and process [32]. Since the facial image is inverted, it breaks the original layout of the face and has a greater impact on the configural processing method [33]. Participants need to reintegrate featural information from various areas of the face, such as eyes and mouth. Therefore, participants have difficulty in recognizing inverted facial images compared to upright facial images. There is a huge contrast in their reactions, known as the inversion effect [34]. If there is an inversion effect, it can be inferred that this participant mainly adopts a configural processing method.

For TD children, inverted faces are more difficult to recognize than upright faces, and there are significant differences between these two facial presentations. TD children are affected by the inversion effect, which is consistent with their predominant use of configural processing. Langdell [35] first used this paradigm and found no inversion effect in children with ASD during the experiment. This conclusion supported that children with ASD did not rely on configural processing. Many researchers supported this conclusion and believed that children with ASD were not affected by the inversion effect, and were more inclined to use a featural processing method [36]. However, some others considered that children with ASD could also be affected by the inversion effect, supporting the conclusion that children with ASD had the capacity of configural processing [37]. Pallett et al. [38] found that with increasing age and IQ, children with ASD would be affected by the inversion effect like TD children. They would be sensitive to faces and encode the facial information using a configural processing method. The controversy in these studies might be due to the heterogeneity of participants and the differences in experimental tasks. Therefore, the impact of the inversion effect on children with ASD remains to be further investigated.

Some researchers thought that a single paradigm experiment could not fully reveal the inner processing mechanism of children with ASD. The combination of spatial frequency and inversion effect might provide new clues about the characteristics of facial expression processing in children with ASD. The findings of Kikuchi et al. [39] showed that children with ASD had an inversion effect under the LSF condition, demonstrating the capacity for configural processing of facial expressions in children with ASD. Furthermore, it is worth in-depth exploring whether children with ASD can fully fixate and acquire facial expression features from the core areas of the face, and whether they can make adaptive adjustments under different conditions.

As reviewed above, researchers generally believe that children with ASD have certain disorders in the processing of facial expressions. There are still some issues worthy of in-depth study on the internal processing mechanism of children with ASD. (1) There is still controversy about what facial expression processing strategies children with ASD use. The combination of multiple experimental paradigms is a beneficial research avenue to reveal their processing mechanisms. It is worth further studying the relationship of different conditions and their influence on the facial expression processing of children with ASD. (2) There is still controversy about the visual processing mechanisms of the face and specific areas of interest in children with ASD. Eye tracking technology is an important tool to reveal their tacit emotional and cognitive processing. It deserves further research on the eye tracking indicators of children with ASD in core areas of the face, such as eyes and mouth, under different conditions, as well as their adjustment strategies affected by different factors. (3) The age of the participants had a wide distribution across experiments. Young children with ASD are in the golden age of brain development and can be effectively improved through intervention. They need to be the subjects of more research.

In view of this, this research is based on previous studies and intends to explore the influence of spatial frequency and inversion effect on facial expression processing of children with ASD through eye tracking technology, so as to further explore their facial expression processing mechanism. This study intends to use comparative experimental research. The experimental group is children with ASD, and the control group is matched TD children. The differences in facial expression processing and eye tracking characteristics between children with ASD and TD children are compared and analyzed. This experiment adopts a multi-factor mixed experimental scheme to analyze the facial expression recognition rates of the two groups of children under different spatial frequencies and orientations, as well as eye tracking indicators for core areas such as eyes and mouth, and their correlation, so as to deeply study the facial expression processing mechanism of children with ASD.

This research uses eye tracking technology and different experimental paradigms to explore the facial expression processing characteristics of children with ASD, and reveal their processing mechanism, which has theoretical significance. On the other hand, it can provide a basis for the design of facial expression intervention materials and their presentation forms, which has practical value for children with ASD.

## 2. Materials and Methods

### 2.1. Participants

The experiment of facial expression processing required participants to have certain abilities in visual attention, cognition, and comprehension. Due to the prevalence of developmental delay in children with ASD, the inclusion criteria for the two study groups were specified as two groups of children with matched abilities rather than matched ages. In addition, children with attention deficit and difficulty completing the experiment were excluded.

The participants consisted of 12 children with ASD from a special education institution in Wuhan (ASD group: 9 males and 3 females; 5–7 years old; mean age = 5.6 years, SD  =  0.5 years) and 11 TD children from a kindergarten in Xinyang (TD group: 7 males and 4 females; 3–5 years old; mean age  =  4.1 years, SD  =  0.3 years). The Peabody Picture Vocabulary Test Revised (PPVT-R) was used to assess their abilities [40,41], and the results were analyzed by *t*-test. It was found that there was no significant difference in the level of verbal IQ between these two groups (ASD group: mean score = 51.08, SD = 14.66; TD group: mean score = 53.54, SD = 4.23; *t* = −0.56, *p* = 0.59 > 0.05), which met the experimental requirements.

Before the experiment, all children with ASD had been double-blindly diagnosed by two expert physicians in child development and behavior, and confirmed the diagnoses according to DSM-5 criteria [42]. After parental interviews and clinical observations, the two groups of participants were excluded from childhood schizophrenia, epilepsy, and other organic brain diseases, and were confirmed to have normal vision (or corrected vision) and normal intelligence.

Privacy protection agreements were signed with the special education institution and the kindergarten. Informed consent was obtained from the participants’ parents. This study only collected relevant data anonymously when the participants completed the experimental tasks. No personally identifiable information or portraits of participants were involved.

### 2.2. Design

A multi-factor mixed experiment of 2 (group) × 3 (spatial frequency) × 2 (orientation) was designed. When analyzing eye tracking data, another factor (area of interest) would be added. The between-subject variable was the group, divided into two levels of ASD group and TD group. The others were within-subject variables. The spatial frequency includes three levels: broad spatial frequency (BSF), which is the original image, low spatial frequency (LSF), and high spatial frequency (HSF). The orientation includes two levels: upright and inverted. The area of interest includes two levels: eyes and mouth.

### 2.3. Materials

In order to obtain high-quality images for facial expression processing, the standardized facial expression datasets were required, with standardized facial angles, expression types, expression strengths, and a rich source of subjects. There were several datasets that satisfied the experimental needs. The formal experimental materials used the BU-4DFE database of State University of New York at Binghamton [43,44], which had been purchased and licensed for non-profit research use. This was a high-resolution facial expression database presenting fine-grained expression structural variation, including multiple ethnicities, a broad age range, more than 100 subjects, 6 basic expressions, each including 4 intensity levels. The facial expression images of 4 young Asians (2 males and 2 females, with an average age of 22 years) were selected as experimental materials. Each person’s facial images contained 4 basic expressions: happiness, sadness, anger, and fear, all of which were facial expressions at the highest intensity level for easy identification by children.

**Figure 1:**
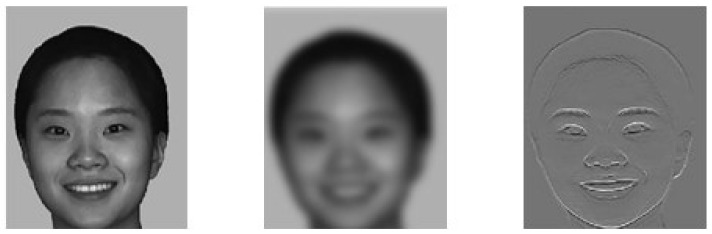
Example of experimental material (From left to right: BSF image, LSF image, HSF image).

Photoshop CS6 software (Adobe, San Jose, CA, USA) was used for normalization and grayscale processing, and then the MATLAB 2018b software (MathWorks, Natick, MA, USA) was used to process the images: first with Fourier transform, and then with Gaussian filter for LSF processing and HSF processing. The filtering standard was international general standard [30]: LSF parameter < 2 cycle/face, HSF parameter > 6 cycle/face. Finally, a total of 48 facial expression images in the upright states were formed, including 16 BSF images of the original image, 16 LSF images, and 16 HSF images. Then these images were rotated by 180° to obtain another 48 facial expression images in the inverted state. Figure 1 was an example of the formal experimental materials.

### 2.4. Equipment

An all-in-one computer with a 23-inch multi-touch screen was used to present the experimental materials on the screen and record the participants’ responses. The screen resolution was 1920 × 1080 pixels.

An Eye Tracker 4C (Tobii, Stockholm, Sweden) was mounted directly below the computer screen, connected to and controlled by this computer. It had a sampling frequency of 90 Hz and offered the software development kit for eye tracking data acquisition. The calibration of the eye tracker followed the standard procedure provided by Tobii device driver, called the 7-point positioning method. That is, the participants were required to gaze at 7 target points on the screen in sequence (a central point, then three peripheral points, then another set of three peripheral points), staring at each point until it disappeared to complete the calibration. Failure to calibrate at any point would result in a recalibration of all 7 points. Only after successful calibrating all 7 points were the participants allowed to take part in further steps of the experiment.

### 2.5. Procedure

The experiment consisted of two blocks, upright and inverted. Six children with ASD and six TD children observed the upright block first, and the others observed the inverted block first. Each block had 48 trials, that is, 48 facial expression images consisting of 3 spatial frequency conditions, from 4 Asians, and 4 basic expressions of each Asian. All these trials were conducted in random order. Each participant was required to complete a total of 96 trails from these two blocks. The experimental task of facial expression processing adopted the two-alternative forced-choice (2FAC) matching task, containing one target image of the facial expression and two probes (matching option and non-matching option).

**Figure 2:**
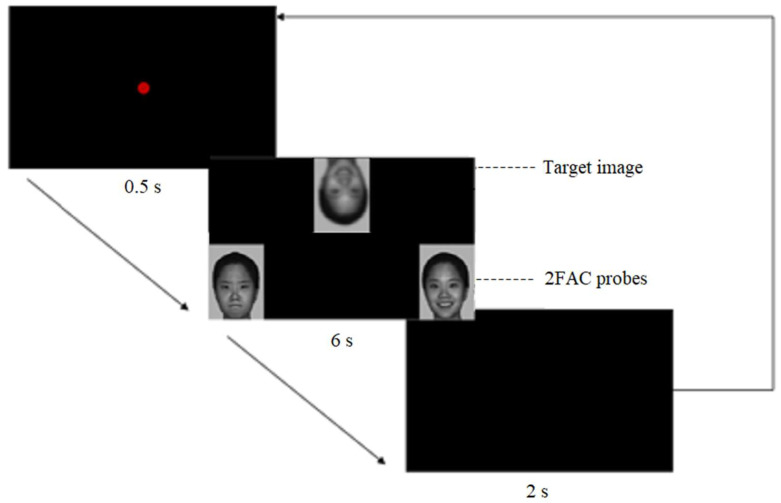
Example of experimental procedure (A red dot was presented for 0.5 s to attract the participant’s attention. Subsequently, the target image and 2FAC probes were presented for 6 s. The participant was required to find the correct probe. Then a black screen appeared for 2 s as a rest).

The procedure was as follows: first, a red dot was presented in the center of the black screen for 0.5 s to attract the participant’s attention. Next, the experimental materials were presented with the target image at the top middle and the 2FAC probes at the bottom of the screen. Each trail lasted for 6 s. The participant was asked to carefully observe the target image and found the correct probe. In the interval between two trails, a black screen appeared for 2 s as a rest. This process was repeated in turn until the block ended. Each block took about 7 min. The participant was allowed to rest for half an hour or more in the interval between two blocks. Figure 2 was an example of the experimental procedure.

The experiment was carried out in a quiet and comfortable room. Each participant was required to sit on a chair 60–65 cm away from the computer screen, and performed the eye tracking calibration. Two operators guided the participant to complete the experimental task. Operator A was responsible for controlling the computer program and eye tracker, presenting each target image on the screen. Operator B prompted the participant to watch the target image and gave instructions to the participant in a language adapted to his/her level, for example, “Hello kid! Look! Which one of the following expressions do you think is the same as the target image?” The participant needed to touch the correct probe or verbalize the expression type within the specified time. The operator would assist the participant in the tasks until the end of the experiment. The computer program automatically recorded the participant’s experimental data, including score, elapsed time and eye tracking data. The experiment adopted the 0/1 scoring method: 1 point for correctness, 0 for errors or no response. When the participant chose the correct probe, a cartoon character would appear on the screen as reward feedback.

### 2.6. Data Analysis Indicators

The experiment recorded the resultant data of the participants completing the experimental task, that is, the facial expression recognition rate, which comprehensively reflected their facial expression processing ability. This article focused on the influence of spatial frequency and inversion effect on facial expression processing of children with ASD and TD children. Statistical analysis was performed using SPSS 27 (IBM, Armonk, NY, USA) to discuss their differences and infer their respective processing methods of facial expressions. The influence of other factors (different characters, different expression types) of the experimental materials would be discussed in another article. The indicator of facial expression recognition rate in this article was the average recognition rate of participants for different characters and different types of expressions.

The eye tracker recorded the procedural data of the participants during the experimental task. Eye tracking indicators included: (1) Fixation count, referred to the total number of fixation points in the target area. (2) Fixation duration, referred to the total duration of fixation on the target area. These indicators were used to explore the facial expression processing characteristics of the two groups of children on target image (face) and specific areas of interest (eyes, mouth). Ogama 5.1 (Opensource software) was used for the division of areas of interest, eye tracking data statistics, and result visualization. The fixation calculation was performed using the default fixation detection algorithm built into the Ogama 5.1 software released from LC technologies. The parameters were also the default values (maximum distance in pixels was 20, and minimum number of samples was 5).

The eye tracking indicators of the two groups of children on the eyes and mouth areas under different conditions reflected the amount of facial expression information they obtain from the target area, which could comprehensively reflect their facial expression processing ability and reveal their tacit emotional and cognitive processing. It could also be inferred whether and how they adaptively adjusted facial expression processing strategies under the influence of different spatial frequencies and inversion effects. Using SPSS for statistical analysis, the correlation between the attention to eyes/mouth area and the facial expression recognition rate was obtained, which is helpful to deeply explore the internal processing mechanism of the two groups of children, and reveal the causes of facial expression processing disorders in children with ASD.

## 3. Results

### 3.1. Facial Expression Recognition Rate

For each condition of spatial frequency and orientation, the mean facial expression recognition rates of the two groups of children and the Mann–Whitney U test results (*p*-values) are shown in Table 1.

The distribution of the facial expression recognition rate made the use of parametric tests inappropriate. Therefore, the Mann–Whitney U test was performed on these data of the two groups of children. Bonferroni corrections were adopted for all comparisons. It could be seen that the facial expression recognition rates of the two groups of children under different conditions were significantly different (*p* < 0.05). That is, the average expression recognition rate of children with ASD under each condition was significantly lower than that of TD children.

The Friedman test results showed that the conditional effect was significant in both groups (*p* < 0.05). Then the Wilcoxon test with Bonferroni correction was performed in each group. The results showed that the facial expression recognition rate of children with ASD under the LSF condition was significantly lower than that under the other two spatial frequency conditions (*p* < 0.01). When the face was upright, the recognition rate of children with ASD under the HSF condition was significantly higher than that under the other two spatial frequency conditions (*p* < 0.05), while the recognition rate of TD children under the BSF condition was significantly higher than that under the other two spatial frequency conditions (*p* < 0.05). When the face was inverted, the recognition rate of TD children under the LSF condition was significantly lower than that under the other two spatial frequency conditions (*p* < 0.05). Additionally, TD children had the inversion effect under all the three spatial frequency conditions, which was manifested as a significant decrease in recognition rate (*p* < 0.05). However, children with ASD were less affected by the inversion effect. They only had the inversion effect under the LSF condition, that is, the recognition rate decreased significantly when the face was inverted (*p* < 0.05). They had no inversion effect under the BSF and HSF conditions (*p* > 0.05).

### 3.2. Eye Tracking Data

#### 3.2.1. Fixation Counts on the Target Image

For each condition of spatial frequency and orientation, the mean fixation counts of the two groups of children on the target image and the *t*-test results (*p*-values) are shown in Table 2.

A repeated measures analysis of variance of 2 (group) × 3 (spatial frequency) × 2 (orientation) was performed on the fixation counts. The results showed that the main effect of the group was significant, F(1,21) = 41.89, *p* = 0.000 < 0.01, R^2^ = 0.67. The main effect of spatial frequency was significant, F(2,42) = 18.45, *p* = 0.000 < 0.01, R^2^ = 0.47. The main effect of orientation was not significant, F(1,21) = 0.06, *p* = 0.80 > 0.05, R^2^ = 0.003. The interaction effect between the group and spatial frequency was significant, F(2,42) = 4.97, *p* = 0.012 < 0.05, R^2^ = 0.19. The interaction effect between the group and orientation was not significant, F(1,21) = 3.55, *p* = 0.07 > 0.05, R^2^ = 0.15. The interaction effect between spatial frequency and orientation was not significant, F(2,42) = 1.05, *p* = 0.36 > 0.05, R^2^ = 0.05. The interaction effect among the group, spatial frequency and orientation were significant, F(2,42) = 4.23, *p* = 0.02 < 0.05, R^2^ = 0.17.

A simple effect analysis of the interaction was carried out and showed that when the face was upright, the fixation counts of children with ASD on the target image under all three spatial frequency conditions were significantly fewer than those of TD children (*p* < 0.05). The fixation counts of children with ASD on the target image under the HSF condition was significantly more than those under the other two spatial frequency conditions (*p* < 0.05), while the fixation counts of TD children on the target image under the LSF condition was significantly fewer than those under the other two spatial frequency conditions (*p* < 0.05). When the face was inverted, the fixation counts of children with ASD on the target image was significantly reduced under the LSF condition (*p* < 0.01) and significantly increased under the HSF condition (*p* < 0.05), while the fixation counts of TD children on the target image was significantly reduced under the BSF condition (*p* < 0.05). The fixation counts of children with ASD on the target image under the LSF condition was significantly fewer than that of TD children (*p* < 0.01).

#### 3.2.2. Fixation Counts on the Areas of Interest

For each condition of spatial frequency and orientation, the mean fixation counts of the two groups of children on different areas of interest (eyes, mouth) in the target image and the *t*-test results (*p*-values) are shown in Table 3.

The proportion of children with ASD’s fixation counts on the eyes area to the target facial image was from 16.5% to 27.0%, and on the mouth area was from 44.0% to 70.0%. The proportion of TD children’s fixation counts on the eyes area to the target facial image was from 34.9% to 70.1%, and on the mouth area was from 15.0% to 27.1%.

A repeated measures analysis of variance of 2 (group) × 3 (spatial frequency) × 2 (orientation) × 2 (AOI) was performed on the fixation counts. The results showed that the main effect of the group was significant, F(1,21) = 34.40, *p* = 0.000 < 0.01, R^2^ = 0.62. The main effect of spatial frequency was significant, F(2,42) = 5.22, *p* = 0.009 < 0.01, R^2^ = 0.20. The main effect of orientation was significant, F(1,21)= 12.99, *p* = 0.002 < 0.01, R^2^ = 0.38. The main effect of AOI was significant, F(1,21) = 12.11, *p* = 0.002 < 0.01, R^2^ = 0.37. The interaction effect between the group and spatial frequency was significant, F(2,42) = 8.13, *p* = 0.001 < 0.01, R^2^ = 0.28. The interaction effect between the group and orientation was not significant, F(1,21) = 1.10, *p* = 0.31 > 0.05, R^2^ = 0.05. The interaction effect between the group and AOI was significant, F(1,21) = 320.92, *p* = 0.000 < 0.01, R^2^ = 0.94. The interaction effect between spatial frequency and orientation was not significant, F(2,42) = 1.50, *p* = 0.24 > 0.05, R^2^ = 0.07. The interaction effect between spatial frequency and AOI was significant, F(2,42) = 5.69, *p* = 0.007 < 0.01, R^2^ = 0.21. The interaction effect between orientation and AOI was not significant, F(1,21) = 2.25, *p* = 0.15 > 0.05, R^2^ = 0.10. The interaction effect among the group, spatial frequency and orientation were not significant, F(2,42) = 0.22, *p* = 0.80 > 0.05, R^2^ = 0.011. The interaction effect among the group, spatial frequency and AOI were significant, F(2,42) = 4.78, *p* = 0.013 < 0.05, R^2^ = 0.19. The interaction effect among the group, orientation and AOI were significant, F(1,21) = 46.86, *p* = 0.000 < 0.01, R^2^ = 0.69. The interaction effect among spatial frequency, orientation, and AOI were not significant, F(2,42) = 0.79, *p* = 0.46 > 0.05, R^2^ = 0.04. The interaction effect among the group, spatial frequency, orientation, and AOI were not significant, F(2,42) = 0.16, *p* = 0.85 > 0.05, R^2^ = 0.01.

A simple effect analysis of the interaction was carried out and showed that the fixation counts of children with ASD on the mouth area under each condition were significantly more than those on the eyes area (*p* < 0.05). However, the fixation counts of TD children on the eyes area under each condition were significantly more than those on the mouth area (*p* < 0.05). In addition, TD children had significantly more fixation counts on the eyes area than children with ASD under each condition (*p* < 0.01). The fixation counts of children with ASD on the mouth area under the HSF condition were significantly more than those of TD children (*p* < 0.05). When the face was upright, the fixation counts of children with ASD on the eyes and mouth area under the HSF condition were significantly more than those under the LSF condition (*p* < 0.05). When the face was inverted, the fixation counts of children with ASD on the mouth area under the HSF condition were significantly more than those under the other two spatial frequency conditions (*p* < 0.05). The fixation counts of TD children on the eyes and mouth area under the BSF condition were significantly more than those under the LSF condition (*p* < 0.05). When the face was inverted, the fixation counts of children with ASD on the mouth area were significantly increased under the HSF condition (*p* < 0.05), while the fixation counts of TD children on the eyes area were significantly increased under all three spatial frequency conditions (*p* < 0.05). Children with ASD had significantly more fixation counts on the mouth area than TD children under all three spatial frequency conditions (*p* < 0.05) when the face was inverted.

#### 3.2.3. Fixation Duration on the Target Image

For each condition of spatial frequency and orientation, the mean fixation duration of the two groups of children on the target image and the *t*-test results (*p*-values) are shown in Table 4.

A repeated measures analysis of variance of 2 (group) × 3 (spatial frequency) × 2 (orientation) was performed on the fixation duration. The results showed that the main effect of the group was significant, F(1,21) = 35.51, *p* = 0.000 < 0.01, R^2^ = 0.63. The main effect of spatial frequency was significant, F(2,42) = 14.29, *p* = 0.000 < 0.01, R^2^ = 0.41. The main effect of orientation was significant, F(1,21) = 6.54, *p* = 0.02 < 0.05, R^2^ = 0.24. The interaction effect between the group and spatial frequency was significant, F(2,42) = 12.08, *p* = 0.000 < 0.01, R^2^ = 0.37. The interaction effect between the group and orientation was significant, F(1,21) = 5.71, *p* = 0.03 < 0.05, R^2^ = 0.21. The interaction effect between spatial frequency and orientation was not significant, F(2,42) = 0.22, *p* = 0.80 > 0.05, R^2^ = 0.01. The interaction effect among the group, spatial frequency, and orientation were not significant, F(2,42) = 0.47, *p* = 0.63 > 0.05, R^2^ = 0.02.

A simple effect analysis of the interaction was carried out and showed that the fixation duration of children with ASD on the target image was significantly reduced under the LSF condition (*p* < 0.05) and significantly increased under the HSF condition (*p* < 0.05). When the face was upright, the fixation duration of children with ASD on the target image under the BSF and LSF conditions was significantly less than that of TD children (*p* < 0.01). When the face was inverted, the fixation duration of children with ASD on the target image under the LSF condition was significantly less than that of TD children (*p* < 0.01). The fixation duration of TD children on the target image was significantly reduced under all the three spatial frequency conditions (*p* < 0.05).

#### 3.2.4. Fixation Duration on the Areas of Interest

For each condition of spatial frequency and orientation, the mean fixation duration of the two groups of children on different areas of interest (eyes, mouth) in the target image and the *t*-test results (*p*-values) are shown in Table 5.

The proportion of children with ASD’s fixation duration on the eyes area to the target facial image was from 10.1% to 21.9%, and on the mouth area was from 29.2% to 74.2%. The proportion of TD children’s fixation duration on the eyes area to the target facial image was from 34.3% to 67.0%, and on the mouth area was from 16.7% to 27.5%.

A repeated measures analysis of variance of 2 (group) × 3 (spatial frequency) × 2 (orientation) × 2 (AOI) was performed on the fixation duration. The results showed that the main effect of the group was significant, F(1,21) = 20.30, *p* = 0.000 < 0.01, R^2^ = 0.49. The main effect of spatial frequency was significant, F(2,42) = 8.39, *p* = 0.001 < 0.01, R^2^ = 0.29. The main effect of orientation was significant, F(1,21) = 14.67, *p* = 0.001 < 0.01, R^2^ = 0.41. The main effect of AOI was not significant, F(1,21) = 0.13, *p* = 0.73 > 0.05, R^2^ = 0.01. The interaction effect between the group and spatial frequency was significant, F(2,42) = 9.99, *p* = 0.000 < 0.01, R^2^ = 0.32. The interaction effect between the group and orientation was not significant, F(1,21) = 0.49, *p* = 0.49 > 0.05, R^2^ = 0.02. The interaction effect between the group and AOI was significant, F(1,21) = 99.73, *p* = 0.000 < 0.01, R^2^ = 0.83. The interaction effect between spatial frequency and orientation was not significant, F(2,42) = 0.32, *p* = 0.73 > 0.05, R^2^ = 0.02. The interaction effect between spatial frequency and AOI was significant, F(2,42) = 13.02, *p* = 0.000 < 0.01, R^2^ = 0.38. The interaction effect between orientation and AOI was not significant, F(1,21) = 1.08, *p* = 0.31 > 0.05, R^2^ = 0.05. The interaction effect among the group, spatial frequency and orientation were not significant, F(2,42) = 0.72, *p* = 0.49 > 0.05, R^2^ = 0.03. The interaction effect among the group, spatial frequency, and AOI were significant, F(2,42) = 6.45, *p* = 0.04 < 0.05, R^2^ = 0.24. The interaction effect among the group, orientation, and AOI were significant, F(1,21) = 35.47, *p* = 0.000 < 0.01, R^2^ = 0.63. The interaction effect among spatial frequency, orientation, and AOI were not significant, F(2,42) = 2.49, *p* = 0.10 > 0.05, R^2^ = 0.11. The interaction effect among the group, spatial frequency, orientation, and AOI were not significant, F(2,42) = 0.74, *p* = 0.48 > 0.05, R^2^ = 0.03.

A simple effect analysis of the interaction was carried out and showed that the fixation duration of children with ASD on the mouth area under each condition was significantly more than that on the eyes area (*p* < 0.05). However, the fixation duration of TD children on the eyes area under each condition was significantly more than that on the mouth area (*p* < 0.05). In addition, TD children had significantly more fixation duration on the eyes area than children with ASD under each condition (*p* < 0.01). The fixation duration of children with ASD on the mouth area under the HSF condition was significantly more than that under the other two spatial frequency conditions (*p* < 0.05). When the face was upright, the fixation duration of children with ASD on the mouth area under the HSF condition was significantly more than that of TD children (*p* < 0.05). When the face was inverted, the fixation duration of children with ASD on the mouth area under the BSF and HSF conditions was significantly more than that of TD children (*p* < 0.05). Under all three spatial frequency conditions, the fixation duration of children with ASD on the mouth area was significantly increased (*p* < 0.05), while the fixation duration of TD children on the eyes area was significantly increased (*p* < 0.05) when the face was inverted.

### 3.3. Eye Tracking Visualization

**Figure 3:**
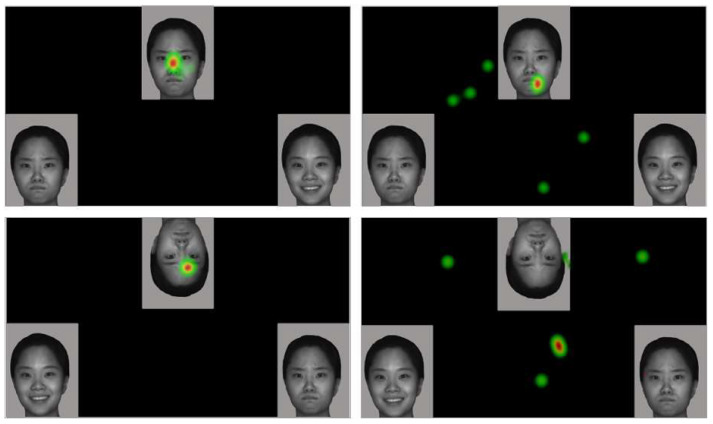
Example of a heat map of eye tracking results (Left: TD children; Right: children with ASD. Colored spots represent areas of concentrated fixation. The redder the color, the more concentrated the fixation in the area. Whereas green represents areas with relatively less fixation).

The Ogama 5.1 (Opensource software, http://www.ogama.net/accessed on 6 January 2022) was used to analyze the eye tracking data of the two groups of children and visualize the results, as shown in Figure 3.

TD children mainly focused their visual attention on the core area of the target face, especially the eyes area. However, children with ASD had more distracted visual attention, and they preferred to stare at the mouth area. The visualized heat map reflected the facial expression processing characteristics of children with ASD and their preference for mouth features.

## 4. Discussion

### 4.1. Overall Analysis

This study explored the differences in facial expression processing and eye tracking features between children with ASD and TD children. The overall results showed that the facial expression recognition rate of children with ASD under various experimental conditions (spatial frequency, orientation) was significantly lower than that of TD children. It could be inferred that the facial expression processing ability of children with ASD was weaker than that of TD children. Due to the prevalence of developmental delay in children with ASD, the participants in this study consisted of two groups of children with no significant difference in the level of verbal IQ. Children with ASD were older than TD children, but their performance in experimental tasks was still significantly weaker than that of TD children, which further indicated that children with ASD had facial expression processing disorders.

The eye tracking results showed that the fixation counts and fixation duration of children with ASD on the mouth area under each condition were significantly more than those on the eyes area. In contrast, the fixation counts and fixation duration of TD children on the eyes area under each condition were significantly more than those on the mouth area. In addition, the fixation counts and fixation duration of TD children on the eyes area under each condition were significantly more than those of children with ASD on the eyes area.

The correlation between the attention to eyes/mouth area and the facial expression recognition rate under each condition was statistically analyzed, and the results are shown in Table 6.

The results of Pearson correlation analysis showed that there was a significant positive correlation between children’s attention (fixation counts and fixation duration) to eyes area and facial expression recognition rates under both BSF and LSF conditions (r > 0.5, *p* < 0.01). Additionally, there was a significant positive correlation between children’s fixation counts on the eyes area and facial expression recognition rate under the condition of inverted face and HSF (r = 0.544, *p* < 0.01). There was a significant positive correlation between children’s fixation duration on the eyes area and facial expression recognition rate under the condition of upright face and HSF (r = 0.505, *p* < 0.05). It could be inferred that children with more visual attention to the eyes area would achieve higher facial expression recognition rates. There was a significant negative correlation between children’s attention (fixation counts and fixation duration) to the mouth area and facial expression recognition rates under the HSF condition (r < −0.4, *p* < 0.05) and under the condition of inverted face and BSF (r < −0.4, *p* < 0.05). It could be inferred that children with more visual attention to the mouth area would achieve lower facial expression recognition rates.

From the comparison results of the two groups of children, it could be inferred that different deployment of visual attention to eyes/mouth area in the two groups of children might lead to different abilities to process and recognize facial expressions. TD children had more visual attention to the eyes and could perceive and acquire relatively more facial expression information, so as to perform relatively more adequate facial expression processing. However, children with ASD preferred features of the mouth area, and lacked visual attention and processing of the eyes area, which might lead to their relatively weaker ability to process and recognize facial expressions than TD children. Therefore, it could be inferred that the facial expression processing disorders of children with ASD were mainly due to their atypical facial expression processing methods and strategies.

The eye avoidance hypothesis provided a certain explanation for why children with ASD had the manifestation of a lack of attention to the eyes [45]. Individuals with ASD perceived the eyes as socially threatening. Direct eye contact would trigger their strong physiological response, such as an increase in skin conductance or amygdala activity [46]. Avoiding the eyes was an adaptive strategy for them.

### 4.2. The Influence of Spatial Frequency on Facial Expression Processing

The change of spatial frequency would cause the change of expression features in the facial image, which would have an impact on different facial expression processing methods. It was generally believed that the low spatial frequency (LSF) was beneficial to the configural processing method. The high spatial frequency (HSF) was beneficial to the featural processing method. Additionally, the broad spatial frequency (BSF) was the original image itself, which contained all the facial information [28]. This study explored the effects of these three spatial frequencies on the facial expression processing of two groups of children. They exhibited different facial expression processing characteristics.

The experimental results showed that the recognition rate of TD children under the BSF condition was significantly higher than that under the other two spatial frequency conditions (*p* < 0.05) when the face was upright. It indicated that rich facial feature information could help TD children to process facial expressions. The change of spatial frequency weakened the facial feature information, affected the visual perception and information processing of TD children, thereby causing difficulties in facial expression recognition [27].

When the face was inverted, the recognition rate of TD children under the LSF condition was significantly lower than that under the other two spatial frequency conditions (*p* < 0.05). Although the LSF condition was beneficial to the configural processing method, TD children also suffer from inversion effects at this time. The eye tracking results showed that the fixation counts of TD children on the target image under the LSF condition were significantly fewer than those under the other two spatial frequency conditions (*p* < 0.05), and the fixation counts of TD children on the eyes and mouth area under the LSF condition were significantly fewer than those under the BSF condition (*p* < 0.05). The decrease in the acquisition of facial information was considered to be an important reason for the decline in their recognition rate. In addition, Deruelle et al. [30] gave a certain explanation, which might be related to their age. The configural processing ability of TD children gradually increased with age. When children were younger, as in the case of this study, their configural processing abilities were weaker. They failed to interpret facial expressions under the LSF condition, resulting in a decrease in facial expression recognition rates.

The experimental results showed that the facial expression recognition rate of children with ASD under the LSF condition was significantly lower than that under the other two spatial frequency conditions (*p* < 0.01). In contrast, children with ASD had the highest recognition rate in the HSF condition, which was significantly higher than that under the other two spatial frequency conditions (*p* < 0.05) when the face was upright. It could be seen that changes in spatial frequency had different effects on children with ASD. The HSF condition with more prominent local features was more conducive to the use of the featural processing method for children with ASD to process facial expressions, which was reflected in the high recognition rate [31]. However, the LSF condition with more blurred facial features prevented children with ASD from using their own processing methods, resulting in a significant decrease in facial expression recognition rate [29].

The eye tracking results showed that under HSF condition, children with ASD significantly increased the fixation counts and fixation duration on the target image, as well as the mouth area. It demonstrated that in response to the changes of spatial frequency, TD children had made certain strategic adjustments to increase visual attention to the core areas under the HSF condition, to enhance the interpretation of facial expressions [47].

These results supported that children with ASD spontaneously adopt the featural processing method to process facial expressions, relied more on local features rather than configural information, and were more accustomed to processing facial information under HSF condition that could enhance local features [48]. It also provided a certain basis for the expression intervention of children with ASD using HSF faces.

Comparing the characteristics of the two groups of children, children with ASD had significantly less fixation duration on the target image than TD children (*p* < 0.01) under the LSF condition, and significantly more fixation counts and fixation duration on the mouth area than TD children (*p* < 0.05) under the HSF condition. It could be seen that there were more differences in facial expression processing methods between the two groups of children, and their adjustment strategies under different spatial frequencies were also different.

### 4.3. The Influence of Inversion Effect on Facial Expression Processing

When the face was inverted, the spatial configuration of the face was affected, and the configural information from various facial areas needed to be reintegrated [34]. It had a greater impact on the configural processing method, resulting in a decrease in the facial expression recognition rate [33]. However, the local features were not affected by the inversion effect and had little effect on individuals who adopt the featural processing method. They could still rely on the local features for facial expression processing. Therefore, the inversion effect could be used to evaluate the relative dependence of individuals on the configural processing method [32].

The results of this study showed that when the face was inverted, TD children had the inversion effect under all three spatial frequency conditions, which was manifested as a significant decrease in expression recognition rate. However, children with ASD were less affected by the inversion effect. They had the inversion effect only under the LSF condition, and no inversion effect under the BSF and HSF conditions. This result was consistent with Kikuchi et al.’s conclusion that the occurrence of inversion effect in children with ASD was related to the spatial frequency of the face [39]. However, Kikuchi et al.’s experimental research only used the behavioral indicator of facial expression recognition rate. This study not only analyzed the recognition rates of the two groups of children under different conditions, but also explored their eye tracking characteristics in core facial areas such as the eyes and mouth, and the correlation between the attention to the eyes/mouth area and the facial expression recognition rate, so as to reveal their internal processing mechanism of facial expressions.

This result was different from the study of Pallett et al. [38]. They found that children with ASD and TD children adopt the same configural processing method, and would also be affected by the inversion effect. The differences in the studies might be due to the differences in participants’ demographic characteristics such as age and verbal IQ levels. Pallett et al. studied adolescents aged 13 to 18 with higher average verbal IQ. However, the children with ASD that participated in this experiment were in the younger age group of 5–7 years old, and their average verbal IQ was also relatively low. It would deserve extended research from a larger age range on how the processing of facial expressions in children with ASD change with age and IQ, and whether they would adopt more configural processing and being affected by the inversion effect like TD children.

Under the LSF condition, the local features were blurred, and the featural processing method commonly used by children with ASD was difficult to use. As a result, under the condition of the upright face and LSF, the facial expression recognition rates of children with ASD were reduced. Whether children with ASD would change the processing strategy and switched to the configural processing method needed further analysis. When the face was inverted, the facial configural information was also affected. Children with ASD needed to reintegrate feature information from the areas such as eyes and mouth to process facial expressions. Compared with the condition of the upright face, children with ASD maintained the fixation counts and fixation duration on the eyes area, and significantly increased the fixation duration on the mouth area, but the facial expression recognition rate was significantly reduced. It meant that children with ASD did have an inversion effect under the LSF condition. It could be inferred that children with ASD might have the capability of configural processing, and might adopt the same configural processing as TD children under the LSF condition. Because children with ASD usually relied more on the featural processing method, they tended not to actively use the configural processing method. Only when the local features were ambiguous or absent, that is, under the condition of inverted face and LSF, children with ASD would use the configural processing method. In this situation, the weak central coherence theory was not applicable.

The eye tracking results showed that when the face was inverted, TD children significantly increased the fixation counts and fixation duration on the eyes area (*p* < 0.05) under all three spatial frequencies. In contrast, ASD children significantly increased the fixation duration on the mouth area (*p* < 0.05) under all three spatial frequencies, and significantly increased the fixation counts on the mouth area (*p* < 0.05) under the HSF condition. Comparing the two groups of children, it was found that children with ASD had significantly more fixation counts on the mouth area than TD children (*p* < 0.05), and significantly less fixation counts and fixation duration on the eyes area than TD children (*p* < 0.05). It could be seen that when the face was inverted, both children with ASD and TD children were able to adjust their processing strategies accordingly. However, their strategies and preferred processing areas were different. TD children could consciously concentrate their visual attention to their preferred eyes area. On the other hand, although children with ASD were less affected by the inversion effect, they would also consciously adjust their strategies and focus their attention on the mouth area, to perceive and acquire more expression information. Therefore, their facial expression recognition rate did not decrease significantly under the BSF and HSF conditions.

## 5. Conclusions

Facial expression processing disorder was one of the core causes of social disorder in children with ASD. In this study, eye tracking technology was used to analyze the facial expression processing methods and eye tracking characteristics of children with ASD and TD children. The influence of spatial frequency and inversion effect on the facial expression processing of children with ASD were explored. The main conclusions of this study were as follows:

1. The facial expression processing ability of children with ASD was significantly weaker than that of TD children, that is, the facial expression recognition rate of children with ASD under various experimental conditions (spatial frequency, orientation) was significantly lower than that of TD children.
2. The facial expression processing disorders of children with ASD were mainly due to their atypical facial expression processing methods and strategies. TD children paid more visual attention to the eyes area. However, children with ASD preferred the features of the mouth area and lacked visual attention and processing of the eyes area, which might lead to their relatively weaker ability to process and recognize facial expressions than TD children.
3. Children with ASD mainly used the featural processing method to process facial expression information. HSF highlighted the local feature information of the face, which was more conducive to the use of the featural processing method for children with ASD, reflected in the increase in visual attention to facial feature areas and the improvement in expression recognition rate.
4. TD children had the inversion effect under all three spatial frequency conditions, which was manifested as a significant decrease in expression recognition rate, indicating that TD children mainly used configural processing method. However, children with ASD only had the inversion effect under LSF condition, indicating that children with ASD had the capacity of configural processing under the LSF condition. Therefore, the weak central coherence theory was not applicable under this condition.
5. When the face was inverted or facial feature information was weakened, both children with ASD and TD children would adjust their facial expression processing strategies accordingly, to increase the visual attention and information processing of their respective preferred processing areas. The fixation counts and fixation duration of TD children on the eyes area increased significantly, while the fixation duration of children with ASD on the mouth area increased significantly.

The results of this study provided theoretical and practical support for facial expression intervention in children with ASD. It is possible to consider using HSF images for early intervention training, and then use LSF images for learning transfer to develop their configural processing ability. Meanwhile, children with ASD need to be guided to increase visual attention and information processing on the eyes area.

In addition, this study also had some shortcomings and needed further improvement. The number of participants in this experiment was similar to previous studies. It is worthwhile to further expand the scale and type in future research. As age increases and abilities improve, whether and when children with ASD have new characteristics that are closer to TD children is also worthy of study. Furthermore, in-depth research will consider the use of electro-skin sensors or EEG devices, combined with eye tracking data.

## Figures and Tables

**Table 1:** Facial expression recognition rate under different conditions.

Group	Orientation	Broad Spatial Frequency (BSF)	Low Spatial Frequency (LSF)	High Spatial Frequency (HSF)
ASD group	Upright	0.48 ± 0.19	0.24 ± 0.14	0.57 ± 0.16
	Inverted	0.45 ± 0.14	0.16 ± 0.08	0.47 ± 0.16
TD group	Upright	0.93 ± 0.08	0.77 ± 0.12	0.82 ± 0.16
	Inverted	0.73 ± 0.17	0.53 ± 0.14	0.66 ± 0.17
*p*-value of Mann–Whitney U test	Upright	0.000	0.000	0.002
	Inverted	0.000	0.000	0.023

**Table 2:** Fixation counts on the target image.

Group	Orientation	Broad Spatial Frequency (BSF)	Low Spatial Frequency (LSF)	High Spatial Frequency (HSF)
ASD group	Upright	6.17 ± 2.58	4.17 ± 2.04	8.75 ± 1.91
	Inverted	7.58 ± 3.03	4.17 ± 2.29	9.41 ± 2.35
TD group	Upright	11.73 ± 2.37	8.45 ± 2.21	11.18 ± 3.16
	Inverted	8.82 ± 3.02	9.72 ± 2.57	10.09 ± 2.54
*p*-value of *t*-test	Upright	0.000	0.000	0.035
	Inverted	0.340	0.000	0.517

**Table 3:** Fixation counts on different areas of interest.

Group	Orientation	Area of Interest (AOI)	Broad Spatial Frequency (BSF)	Low Spatial Frequency (LSF)	High Spatial Frequency (HSF)
ASD group	Upright	Eyes	1.67 ± 1.72	1.00 ± 0.85	2.17 ± 1.12
		Mouth	2.83 ± 1.40	1.83 ± 1.34	4.08 ± 1.83
	Inverted	Eyes	1.25 ± 1.76	0.83 ± 0.93	1.75 ± 1.05
		Mouth	3.33 ± 0.49	2.92 ± 1.78	5.75 ± 1.42
TD group	Upright	Eyes	4.09 ± 0.94	4.36 ± 1.86	4.18 ± 1.66
		Mouth	3.18 ± 1.40	1.82 ± 1.66	2.00 ± 1.41
	Inverted	Eyes	5.45 ± 0.93	6.82 ± 1.67	5.73 ± 1.95
		Mouth	2.36 ± 1.12	1.45 ± 0.93	1.91 ± 1.22
*p*-value of *t*-test	Upright	Eyes	0.000	0.000	0.002
		Mouth	0.641	0.983	0.006
	Inverted	Eyes	0.000	0.000	0.000
		Mouth	0.020	0.024	0.000

**Table 4:** Fixation duration on the target image (unit: second).

Group	Orientation	Broad Spatial Frequency (BSF)	Low Spatial Frequency (LSF)	High Spatial Frequency (HSF)
ASD group	Upright	1.19 ± 0.46	0.80 ± 0.24	1.82 ± 0.76
	Inverted	1.27 ± 0.48	0.71 ± 0.27	1.80 ± 0.52
TD group	Upright	1.92 ± 0.40	1.77 ± 0.43	1.92 ± 0.42
	Inverted	1.57 ± 0.31	1.51 ± 0.28	1.46 ± 0.27
*p*-value of *t*-test	Upright	0.001	0.000	0.707
	Inverted	0.087	0.000	0.070

**Table 5:** Fixation duration on different areas of interest (unit: second).

Group	Orientation	Area of Interest (AOI)	Broad Spatial Frequency (BSF)	Low Spatial Frequency (LSF)	High Spatial Frequency (HSF)
ASD group	Upright	Eyes	0.24 ± 0.18	0.16 ± 0.11	0.23 ± 0.17
		Mouth	0.42 ± 0.22	0.23 ± 0.16	0.75 ± 0.40
	Inverted	Eyes	0.28 ± 0.21	0.15 ± 0.08	0.18 ± 0.16
		Mouth	0.67 ± 0.36	0.47 ± 0.25	1.33 ± 0.65
TD group	Upright	Eyes	0.66 ± 0.32	0.75 ± 0.29	0.71 ± 0.30
		Mouth	0.32 ± 0.29	0.38 ± 0.25	0.40 ± 0.33
	Inverted	Eyes	0.97 ± 0.36	1.00 ± 0.29	0.89 ± 0.38
		Mouth	0.32 ± 0.20	0.35 ± 0.20	0.40 ± 0.19
*p*-value of *t*-test	Upright	Eyes	0.001	0.000	0.000
		Mouth	0.367	0.119	0.033
	Inverted	Eyes	0.000	0.000	0.000
		Mouth	0.010	0.238	0.000

**Table 6:** The correlation between the attention to eyes/mouth area and the facial expression recognition rate.

Area of Interest (AOI)	Orientation	Spatial Frequency	Fixation Counts		Fixation Duration	
			Pearson Correlation	Sig. (2-Tailed)	Pearson Correlation	Sig. (2-Tailed)
Eyes	Upright	BSF	0.589 **	0.003	0.532 **	0.009
		LSF	0.688 **	0.000	0.708 **	0.000
		HSF	0.367	0.085	0.505 *	0.014
	Inverted	BSF	0.528 **	0.010	0.550 **	0.007
		LSF	0.793 **	0.000	0.692 **	0.000
		HSF	0.544 **	0.007	0.218	0.318
Mouth	Upright	BSF	0.000	1.000	−0.320	0.136
		LSF	−0.100	0.651	0.398	0.060
		HSF	−0.679 **	0.000	−0.462 *	0.026
	Inverted	BSF	−0.427 *	0.042	−0.485 *	0.019
		LSF	−0.409	0.053	−0.215	0.325
		HSF	−0.423 *	0.044	−0.595 **	0.003

** Correlation is significant at the 0.01 level (2-tailed). * Correlation is significant at the 0.05 level (2-tailed).

## Data Availability

The experimental data generated during the study are not publicly available due to privacy and ethical restrictions. The BU-4DFE database was obtained from the Research Foundation of State University of New York and are available from Professor Lijun Yin with the permission of the Research Foundation of State University of New York.
